# Quality of life among epileptic patients in Khartoum State neurological clinics, Sudan: a cross-sectional study

**DOI:** 10.1097/MS9.0000000000003535

**Published:** 2025-07-09

**Authors:** Doaa Rabeie Hassan AbdEldaim, Rabeie Hassan AbdEldaim Bashir, Siham Ahmed Balla, Shaima Omer Mohamed Elawad, Ola Dafaalla Mohamed Hag Ali, Lina M Omer, Rooa Mohammed, Noor Salaheldin Humaida Elfaki, Sara Elawad, Ahmed Balla M. Ahmed

**Affiliations:** a Faculty of Medicine, University of Khartoum, Khartoum, Sudan; b Department of Medicine, Military Hospital, Omdurman, Sudan; c Department of Medicine, Aliaa Specialist Hospital, Omdurman, Sudan; d Community Medicine Department, Faculty of Medicine, University of Khartoum, Khartoum, Sudan

**Keywords:** cross-sectional study, epilepsy, neurological disorder, quality of life, Short-Form Health Survey (SF-36) model, Sudan

## Abstract

**Background:**

Epilepsy is a chronic non-communicable disease defined as recurrent and unprovoked seizures. Epilepsy causes a wide range of challenges that affect the patient’s quality of life (QoL). This study aimed to assess the quality of life and associated factors among patients with epilepsy in Khartoum State, Sudan.

**Methods:**

This hospital-based cross-sectional study involved epileptic patients recruited from three neurological clinics located in Khartoum State, Sudan. The questionnaire consisted of the Short-Form Health Survey (SF-36) to assess quality of life, along with items covering the patients’ sociodemographic and clinical characteristics. The data were analyzed using SPSS v.23. The Kruskal–Wallis test, Mann-Whitney U test, and multiple logistic regression were used to analyze associations between clinical and demographic factors and QoL scores. The level of significance was set at 0.05.

**Results:**

A total of 106 epileptic patients were interviewed. The total mean score of QoL SF-36 in these patients was 59.8 ± 13.9. A total of 44.3% of the epileptic patients demonstrated a good quality of life. For different SF-36 subscales, the highest mean score was for the physical functioning domain (78.7 ± 21.3). All demographic and clinical characteristics were not significantly associated with the QoL score. Age, gender, and the presence or absence of workdays were found to be significantly associated with the physical functioning subscale (*P* = 0.003, 0.036, and 0.003, respectively), while marital status and number of outpatient visits were significantly associated with the mental health subscale (*P* = 0.041 and 0.038, respectively). Additionally, the presence or absence of workdays was significantly associated with the role limitation due to the physical health subscale (*P* = 0.023).

**Conclusion:**

The study findings suggested that most patients with epilepsy have a moderate-to-good quality of life. The highest score was for the physical functioning domain. A significant relationship was identified between specific socio-demographic and clinical factors and various SF-36 QoL subscales. These findings highlight the need for targeted, culturally sensitive interventions, as well as longitudinal research to address the broader determinants of quality of life in this population.

## Introduction

Epilepsy is a chronic non-communicable disease characterized by recurrent, unprovoked seizures caused by sudden, brief, and excessive electrical discharges in clusters of neurons across various brain regions. The clinical presentation of seizures is influenced by the location of seizure onset, the size of the affected brain region, and the frequency of seizures^[1]^. Epilepsy accounts for over 0.5% of the global disease burden. People with epilepsy often experience comorbidities, including anxiety and depression, as well as physical injuries and intellectual disabilities^[2]^. In Sudan, data on the burden of epilepsy remain scarce. Only two studies have assessed the prevalence of epilepsy, both among children in Khartoum State. A study conducted in 1983 reported a prevalence of 0.9 per 1000 among school-aged children in Khartoum Province^[3]^. A more recent study in 2016 found a higher prevalence of 4 per 1000 among 74 949 school children in Khartoum State^[4]^. However, no epidemiological studies have been conducted among the adult population. Despite the limited data, epilepsy is estimated to account for 1.6% of annual mortality and 238.7 disability-adjusted life years (DALYs) per 100 000 population in Sudan^[5]^.

**HIGHLIGHTS**

- Patients with epilepsy in Khartoum State exhibit a moderate-to-good quality of life.
- The physical functioning domain had the highest QoL score among SF-36 subscales.
- Age, gender, and workdays were linked to physical functioning, while marital status and outpatient visits influenced mental health.
- Further qualitative research is needed to explore predictors of QoL in epileptic patients.

The diagnosis of epilepsy requires a patient to have two or more unprovoked seizures. The type of epilepsy is determined through a detailed medical history, neurological examination, EEG results, and brain imaging such as CT or MRI. Based on these findings, an appropriate treatment strategy and evaluation plan is developed for the patient^[6]^.

Epilepsy is classified into three levels: (i) seizure type, (ii) epilepsy type, and (iii) epilepsy syndrome. Epilepsy syndrome refers to a collection of characteristics that tend to appear together, including seizure types, EEG, and imaging features^[7]^. While understanding the classification and management of epilepsy is crucial for effective treatment, it is equally important to go beyond this medical focus to explore how the condition affects patients’ quality of life.

The World Health Organization defines quality of life as “The way a person views their place in life in relation to their objectives, standards, expectations, and concerns, as well as the culture and value systems in which they live”^[8]^. Epilepsy has profound effects on individuals’ lives, contributing to significant physical, psychological, and social challenges^[9,10]^. Seizures, which are unpredictable, can lead to physical risks such as burns, falls, and drowning, leaving patients feeling insecure and vulnerable^[11]^.

In addition to these physical risks, epilepsy is strongly associated with social stigma and a lower quality of life (QoL)^[12]^. Epileptic patients are more likely to experience depression, anxiety, and low self-esteem. They also face social difficulties, such as lower marriage rates, increased social isolation, and a higher likelihood of being underemployed or unemployed^[9,13]^. Furthermore, the financial burden of epilepsy, including high socioeconomic and medication costs, significantly affects their quality of life^[14]^. These challenges may even increase the risk of suicide among individuals suffering from epilepsy^[15]^.

Together, these interconnected physical, psychological, and socioeconomic factors highlight the profound and multifaceted impact of epilepsy on quality of life.

Today, QoL assessments are becoming increasingly popular as they can provide insight into epilepsy-related complaints of learning, attention, physical pain, and health-related QoL^[16]^. Healthcare planners are realizing more and more that disease measures alone do not adequately determine a person’s state of health^[17]^. A deeper comprehension of QoL of individuals with epilepsy who are living in different countries is crucial, as numerous cultural, ethnic, and economic distinctions influence QoL^[18]^.

While many studies evaluate QoL of epileptics worldwide, there are few comparable studies from developing countries, particularly Sudan. Determining the extent of the issue is essential to the methodical approach to managing epilepsy difficulties and suggesting several approaches to improve it^[19]^. Thus, the purpose of this study was to assess the QoL of patients with epilepsy and its relationship with their socio-demographic and clinical characteristics in Khartoum State, Sudan.

## Methods

This descriptive, cross-sectional, hospital-based study was conducted from 5 September to 13 October 2022 in three neurology clinics located in Khartoum State. The selected clinics included two governmental hospitals, one in Khartoum locality and the other in Omdurman locality, as well as one private hospital in Khartoum locality. These centers were chosen due to their high patient visit frequency. Data were collected from 106 Sudanese epilepsy patients attending follow-up visits. These patients were included through total coverage sampling of all eligible epilepsy cases based on the study’s inclusion criteria during the data collection period. The study included male and female patients aged 18 years or older, while severely ill patients and those presenting with active convulsions were excluded.

Data were collected through face-to-face interviews using a structured questionnaire that included both closed and open-ended questions. The questionnaire consisted of two sections: the first focused on socio-demographic and clinical information such as age group, gender, marital status, occupation, residence, workdays, health insurance, number of outpatient and hospital visit, number of doctors consulted, number of drugs used, and healthcare fees. The second section assessed quality of life using the standardized Short Form-36 (SF-36) model^[20]^. The SF-36 provides raw scores ranging from 0 to 100 across 36 items, divided into nine health subscales: physical functioning (PF) (10 items), role limitations due to emotional problems (RE) (3 items), role limitations due to physical problems (RP) (4 items), social functioning (SF) (2 items), bodily pain (BP) (2 items), health change (1 item), emotional well-being (5 items), energy/vitality (VT) (4 items), and general health (GH) (5 items). The SF-36 is easy to use, well-accepted by patients, and meets high standards of reliability and validity. Its reliability is supported by a Cronbach’s alpha exceeding 0.85, with reliability coefficients above 0.75 across all dimensions, except for the social functioning subscale^[21]^.

The physical aspect of QoL is reflected in the PF, RP, and BP subscales and is associated with the GH subscale, while the mental aspect is reflected in the RE, SF, and emotional well-being subscales and is associated with the VT subscale^[22]^. Unlike other studies, this study included and scored the health change subscale alongside the other eight subscales^[21]^. Scoring involves a multi-step process. First, item responses are recorded according to standardized scoring algorithms to ensure that higher scores uniformly reflect better health status. This is essential, as some items are phrased positively while others are phrased negatively. For example, a low score on an item about limitations may indicate better health, while a high score on an item about vitality indicates better health. Recoding adjusts for this directionality. Following recording, items within each subscale are summed and averaged, then transformed linearly to a 0–100 scale, where 0 represents the poorest health and 100 indicates the best possible health in that domain. This allows comparability across subscales despite differing numbers of items^[23]^.

As an alternative approach to assess overall quality of life, the average of the nine subscale scores was calculated and categorized into four levels: poor (0 to 40), moderate (more than 40 to 60), good (more than 60 to 80), and excellent (more than 80 to 100). The study followed the updated STROCSS 2025: Strengthening the Reporting of cohort, cross-sectional and case-control studies in Surgery guidelines^[24]^.

Data were entered and analyzed using IBM SPSS Statistics version 23. Descriptive statistics were employed to summarize the socio-demographic and clinical characteristics of participants. Categorical variables were presented as frequencies and percentages, while continuous variables (SF-36 scores) were reported as means and standard deviations. The normality of continuous data, particularly SF-36 scores, was assessed using both the Kolmogorov-Smirnov and Shapiro-Wilk tests, which indicated a non-normal distribution.

Accordingly, non-parametric tests were applied where appropriate. The Kruskal–Wallis test was used to compare total QoL scores across multivariate data, while the Mann–Whitney U test was employed for comparisons between two groups. To identify potential predictors and adjust for confounding factors, a multiple linear regression model was constructed using the total SF-36 score as the dependent variable. Predictor variables included relevant demographic and clinical factors such as age, gender, marital status, number of outpatient and hospital visits, number of antiepileptic drugs, health insurance, and workdays. Additionally, correlations between individual QoL subscales and patient characteristics were explored using Kruskal–Wallis and Mann–Whitney U tests, depending on the nature and distribution of the data. A *P* value of <0.05 was considered statistically significant.

## Results

### Socio-demographic and clinical characteristics of epileptic patients

A total of 106 epileptic patients participated in the study. The majority were male (58.5%), with the 18–29 age group being the most represented (35.8%). Over half of the participants were married (51.9%). In terms of occupation, the largest group comprised housewives (18.9%), followed closely by freelancers (18%). Most patients resided in Omdurman City (33.9%), followed by Khartoum City (30.2%) (Table 1).

**Table 1:** Socio-demographic characteristics of epileptic patients, n = 106

Variable	Category	Frequency	Percentage (%)
Age group	18–29 years old	38	35.8
	30–49 years old	33	31.1
	50 years old and above	35	33
Gender	Male	62	58.5
	Female	44	41.5
Marital status	Married	55	51.9
	Single	51	48.1
Occupation	Housewife	20	18.9
	Military soldiers	18	17
	Student	14	13.2
	Employer	18	17
	Freelancers	19	18
	Retired	17	16
Residence	Omdurman	36	33.9
	Khartoum	32	30.2
	Bahri	15	14.2
	Others a	23	21.6

^a^ Aljazeera, Kurdofan, Shendi, Atbara, Blue Nile, Neyala, and New Halfa.

Regarding workdays, 45.3% had no workdays, while 54.7% worked at least two days per week. However, 76.4% had health insurance. In the past month, the total direct healthcare cost per person ranged from 0 SDG (covered by health insurance) to 220 000 SDG, with a mean of 54 331.6 SDG.

Regarding clinical characteristics of the patients, 38.7% of participants had no outpatient visit in the past month, while 43.4% had one visit, and only 1.9% had four visits. In terms of hospital visits, 76.4% had none, and just 0.9% had three visits. As for doctor consultations, 0.9% saw no doctors, 63.2% consulted one, 26.4% saw two, and 0.9% consulted six doctors. In terms of medication use, 67% used one drug, 24.5% used two, 7.5% used three, and 0.9% used five drugs during the past month.

### Quality of life of epileptic patients

The overall mean SF-36 score among epileptic patients was 59.8 ± 13.9. Among the SF-36 subscales, the physical functioning domain recorded the highest mean score (78.7 ± 21.3), while the general health domain had the lowest mean score (31.8 ± 16.4) (Table 2).

**Table 2:** Total SF-36 scale and its subscales score in epileptic patients, n = 106

Scale	Mean	SD	Minimum	Median	Maximum
Physical functioning	78.7	21.3	5	85	100
Role limitation due to emotional problem	77.9	24.9	0	75	100
Role limitation due to physical health	63.5	30.8	0	68.8	100
Social functioning	72.4	20.8	12	75	100
Pain	56.7	31.7	0	57.5	100
Health change	56.4	22.9	25	75	75
Emotional well-being	52.6	15.3	25	50	85
Energy/vitality	41.7	12.5	12.5	43.8	68.8
General health	31.8	16.4	10	35	85
Total	59.8	13.9	26.9	60.3	86.7

**Figure 1.**
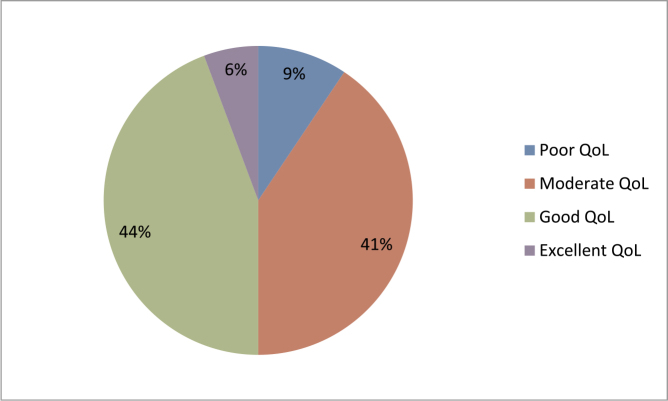
Levels of quality of life of epileptic patients, n = 106.

The study revealed that the majority of epileptic patients (44.3%, n = 47) had a good quality of life. Additionally, 40.6% (n = 43) exhibited a moderate quality of life, 9.4% (n = 10) reported a poor quality of life, and only 5.7% (n = 6) achieved an excellent quality of life (Fig. 1).

### Correlation analysis

The analysis revealed no significant associations between the QoL score and any of the demographic or clinical characteristics, including age, gender, marital status, workdays, health insurance, number of outpatient visits in the last month, hospital visits in the last month, doctors consulted in the last month, number of antiepileptic drugs taken in the last month, and total healthcare fees for the last month, as assessed using the Mann-Whitney test and Kruskal–Wallis test (Table 3).

**Table 3:** Relationships between total QoL-SF36 score and demographic and clinical characteristics, n = 106

Characteristic	Means	Test statistic (H or U)	*P* value
Age^b^		H = 5.861	0.053
18–29	62.69		
30–49	61.27		
50 years and above	55.26		
Gender^a^		U = 1203.0	0.30
Male	60.86		
Female	58.29		
Marital status^b^		H = 2.265	0.519
Single	61.24		
Married	58.97		
Divorced	59.72		
Widowed	48.59		
Workdays^a^		U = 1663.5	0.085
Had workdays	62.00		
Didn’t have workdays	57.14		
Health insurance^a^		U = 1128.0	0.39
Insured	60.40		
Uninsured	57.84		
Number of outpatient visits^b^		H = 3.921	0.207
None	60.75		
Once	59.28		
Twice	55.90		
More than two times	67.58		
Number of hospital visits^b^		H = 4.828	0.185
None	61.37		
Once	54.28		
Twice	55.90		
More than two times	60.14		
Number of doctors consulted^b^		H = 1.998	0.736
One	60.72		
Two	58.80		
Three	59.25		
More than three	52.85		
Number of AED^b^		H = 2.029	0.278
Monotherapy	60.82		
Polytherapy	57.71		
Total healthcare fees^b^		H = 5.621	0.229
0–10 000	56.79		
10 001–50 000	63.39		
50 001–100 000	61.88		
100 001–200 000	54.93		
>200 000	63.10		

^a^ Mann–Whitney test (U).

^b^ Kruskal–Wallis test (H).

Further evaluation using multiple linear regression to determine the adjusted effects of these clinical and demographic factors also yielded a statistically insignificant model (*P* = 0.097), providing insufficient evidence to reject the null hypothesis (Table 4).

**Table 4:** Multiple linear regression model to assess the adjusted effect of epilepsy patients’ factors on their QoL score, n = 106

Factors	Unstandardized coefficients		Standardized coefficients		
	B	Std. error	Beta	t	*P* value
(Constant)	64.4	6.106		10.545	0.00
Marital status	.034	3.118	.001	.011	0.99
Gender	2.517	2.869	.090	.877	0.38
Age in years	−.169	.090	−.222	−1.871	0.06
Total healthcare fee in the past month	3.736E-5	.000	.147	1.370	0.17
Health Insurance	5.163	3.489	.159	1.480	0.14
Number of outpatient visits in the past month	.449	1.593	.029	.282	0.78
Number of hospital visits in the past month	−3.353	2.385	−.137	−1.406	0.16
Number of doctors consulted for epilepsy in the past month	−2.487	1.466	−.170	−1.696	0.09
Number of drugs used in the past month	−1.644	1.954	−085	−.842	0.40
Number of workdays in the past month	.644	.625	.107	1.031	0.31
Model R^2;^ significance	150; F = 1.68; *P* = 0.097				

A correlation analysis was conducted to examine the associations between the demographic and clinical characteristics of the patients and QoL SF-36 subscales. These subscales included Physical Functioning, Role Limitation due to Emotional Problems, Health Change, General Health, Social Functioning, Vitality, Mental Health, Bodily Pain, and Role Limitation due to Physical Health.

Significant findings are summarized in Table 5. Age, gender, and the presence or absence of workdays in the last month were significantly associated with the Physical Functioning subscale (*P* = 0.003, 0.036, and 0.003, respectively). Marital status and the number of outpatient visits in the last month showed significant associations with the Mental Health subscale (*P* = 0.041 and 0.038, respectively). Additionally, the presence or absence of workdays was significantly linked to the Role Limitation due to Physical Health subscale (*P* = 0.023).

**Table 5:** Significant correlations between patients’ personal and clinical data with different QOLIE subscales. n = 106

Variable (subscale)	Patient characteristic	Groups compared	Test statistic (H or U)	*P* value
Physical functioning	Age^a^	18–29, 30–49, and 50+	H = 11.73	0.003^c^
Physical functioning	Gender^b^	Male female	U = 1038.5	0.036^c^
Mental health	Marital status a	Single, married, divorced, and widowed	H = 8.24	0.041^c^
Physical functioning	Workdays^b^	No workdays	U = 1849.0	0.003^c^
		Had workdays		
Role limitation due to physical health	Workdays^b^	No workdays	U = 1742.5	0.023^c^
		Had workdays		
Mental health	Number of outpatient visits^a^	None, once, twice, and more than twice	H = 8.45	0.038^c^

^a^ Kruskal–Wallis test (H).

^b^ Mann–Whitney test (U).

^c^ Significant association at *P* = 0.05. QOLIE, quality of life in epilepsy.

## Discussion

Epilepsy is not only a neurological condition but also a significant determinant of patients’ overall well-being, influencing various aspects of their physical, emotional, and social lives. This study aimed to assess the quality of life among epileptic patients in Khartoum State’s neurological clinics, providing insights into the factors that shape their experiences and outcomes. Improving quality of life is a primary goal of epilepsy treatment^[25]^, as it encompasses more than just seizure control – it addresses the broader impact of the condition on daily functioning and social integration. Previous studies have shown that epileptic patients in the Middle East^[26]^ and Africa^[26,27]^ face significant sociocultural and healthcare disadvantages compared to their counterparts in Western societies^[28,29]^. Despite these challenges, there remains a scarcity of QoL research from these regions, underscoring the importance of exploring this issue to inform culturally appropriate interventions and improve patient outcomes.

Various socio-demographic and clinical characteristics were assessed in this study to detect their possible impact on quality of life. The age range of the participants was 18–85 years, with 64.2% aged 30 years and above, and more than half of them were male 58.5% in alignment with the study conducted in Ethiopia^[30]^. Half of the participants were married 51.9%, and 52% of them had an occupation; this is a good percentage compared to the fact that epileptic patients are at greater risk of problems concerning marriage and employment status^[31]^.

To assess quality of life, researchers typically rely on direct patient interviews or standardized questionnaires^[32]^. In this study, the overall quality of life among epileptic patients, as measured by the SF-36 scale, was moderate. This finding is consistent with studies conducted in Ethiopia using the WHOQOL-BREF scale and in rural Kenya, both of which reported similar results^[33,34]^. However, these outcomes differ from a study in Iraq, where patients reported substantially lower QoL scores using the SF-36 survey^[35]^. These variations may be attributed to differences in healthcare accessibility, cultural perceptions of epilepsy, or social and economic conditions.

Regarding the SF-36 subscales, physical functioning, role limitation due to the emotion domain, and social functioning had the highest scores, indicating better performance in these areas. This suggests that despite living with epilepsy, many patients can maintain reasonable physical capacity, social interactions, and emotional roles, likely due to family or community support systems. These findings align with studies in the Netherlands and Canada, where similar domains scored the highest^[36,37]^. Moreover, the physical and social domain scores in our study were higher than those reported in a previous Sudanese study conducted in 2009^[38]^. That earlier study was carried out in three cities – Khartoum, Wad Medani, and Atbara – while our study was limited to Khartoum, the capital. This difference may reflect better access to healthcare services, stronger social support networks, and improved public awareness in the capital city compared to other regions. The exclusion of other cities may have contributed to the higher scores observed in our findings.

Conversely, the general health domain recorded the lowest scores, highlighting patients’ ongoing concerns about their overall health and well-being. This result is comparable to findings from Iraq, suggesting that the perception of general health remains a challenge for epileptic patients across various settings^[35]^.

Previous studies on QoL among individuals with epilepsy have consistently reported that their QoL is lower than that of the general population^[1,18,37]^. However, our findings revealed that only 9.4% of patients had poor QoL, while approximately 40.6% had moderate QoL. This discrepancy may be explained by differences in the age distribution, sample size, and geographical context of the study. QoL in epileptic patients is influenced by numerous factors, including seizure frequency, which is itself affected by patient compliance, access to antiepileptic drugs, and the quality of medical care. Patients with uncontrolled seizures tend to have significantly lower QoL scores^[39]^.

Our data showed that age, gender, marital status, and workdays had no significant impact on the overall QoL. This demonstrates the contradictory information about the association between QoL and many sociodemographic factors, including age, education level, marital status, employment status, and some clinical characteristics of epileptic patients^[32]^. Additionally, we found that the number of antiepileptic drugs used was not associated with the quality of life score, this is consistent with a previous study which found that the treatment modality had no impact on QoL^[16]^ and contrasts the other which suggested that the use of polytherapy is correlated with lower QoL^[9]^.

In previous studies, age has been highlighted as a significant determinant of QoL in epilepsy. For instance, a study in South Korea by Choi-Kwon *et al* identified age as an important factor influencing QoL^[40]^. However, other researches suggest that older individuals may not always experience poorer QoL compared to younger patients^[41,42]^. In this study, age was significantly correlated with the physical functioning subscale, aligning with previous findings that older adults are more likely to experience reduced physical capacity, fatigue, and lower energy levels^[41]^.

Interestingly, our study found that younger patients reported slightly lower QoL scores. This could be attributed to a recent diagnosis of epilepsy combined with challenges in proper medication management. Another study, however, had shown no significant differences in overall QoL between older and younger patients^[43]^.

The observed association between gender and physical functioning in this study is consistent with findings from previous research. A study conducted in India reported that females generally have worse quality of life compared to males^[44]^, due to biological and social factors. Women often face a higher burden of physical health issues such as fatigue and musculoskeletal conditions, coupled with societal roles that increase their caregiving responsibilities, which can negatively impact their physical well-being. These disparities highlight the need for gender-sensitive approaches in healthcare to address the unique challenges faced by women and improve their quality of life.

Additionally, the mental health subscale was significantly associated with marital status and the number of outpatient visits in the past month. Married individuals generally report greater life satisfaction and better psychological health, which may be due to the support provided by their spouses in managing their condition^[45]^. Outpatient visits often serve as a proxy for the severity of a condition or access to healthcare. Frequent visits may indicate poorer mental health due to chronic health issues or psychological stress related to the condition. Conversely, individuals with fewer or no visits might report better mental health, as they may have less severe conditions or better coping mechanisms. Patients with epilepsy often require frequent and long-term follow-up care, which can significantly impact various aspects of their quality of life domains. Furthermore, a significant correlation was identified between the presence or absence of workdays and the subscales assessing role limitations due to physical health and physical functioning. This may be attributed to the impact of seizures, which can drain energy levels. Even when seizures are fully controlled with medication, side effects such as fatigue or drowsiness can hinder or prevent individuals from carrying out daily tasks effectively.

This study, however, has certain limitations. First, as a cross-sectional study, it lacked a follow-up component, making the results prone to recall bias. Second, important disease-related factors, such as severity, seizure frequency, comorbid psychiatric conditions, and access to treatment, were not examined or controlled for, which may have influenced the reported quality of life outcomes.

## Conclusion

Our study concluded that the majority of patients with epilepsy exhibit a moderate to good QoL compared to the general population, which contrasts with findings from other studies. Only a small proportion of patients were identified as being at risk for poor QoL. The highest scores were observed in the physical functioning domain while the general health domain had the lowest score.

Additionally, the study found no significant association between overall QoL scores and socio-demographic or clinical characteristics of epileptic patients. However, a significant relationship was identified between specific socio-demographic and clinical factors and various SF-36 QoL subscales. We recommend implementing culturally appropriate interventions aimed at improving the general health perception among epileptic patients, enhancing access to consistent outpatient care, and addressing gender-specific needs, particularly for women who may experience lower physical functioning. Efforts should also focus on supporting younger patients in managing new diagnoses and treatment plans, as well as promoting employment opportunities for individuals with epilepsy to enhance their physical and social functioning. Additionally, further longitudinal studies are warranted to assess the impact of seizure frequency, treatment adherence, and comorbidities on quality of life and to guide evidence-based policies and clinical practices tailored to the Sudanese context.

## Data Availability

The datasets generated during and/or analyzed during the current study are available from the corresponding author on reasonable request.

## References

[1] World Health Organisation. Fact sheet. Revised 2024. Available at: https://www.who.int/news-room/fact-sheets/detail/epilepsy (Accessed 12 Sep 2024).

[2] Epilepsy – World Health Organization (WHO). Available at: https://apps.who.int/gb/ebwha/pdf_files/EB146/B146_12-en.pdf (Accessed 11 March 2024).

[3] Younis YO. Epidemiology of epilepsy among school populations in Khartoum Province, Sudan. J Trop Med Hyg 1983;86:213–16. 6672229

[4] Mohamed IN Elseed MA Hamed AA. Prevalence of epilepsy in 74,949 school children in Khartoum State, Sudan. Paediatr Int Child Health 2017;37:188–92. 28162058 10.1080/20469047.2016.1278110

[5] Awad Bashir MB Cumber SN. The quality of life and inequalities in health services for epilepsy treatment among patience in the urban cities of Sudan. Pan Afr Med J 2019;33. 10.11604/pamj.2019.33.10.15440 PMC6607452 31303955

[6] Diagnosis In:Epilepsy Foundation. Available at: https://www.epilepsy.com/diagnosis (Accessed 17 March 2024).

[7] Scheffer IE Berkovic S Capovilla G. ILAE classification of the epilepsies: position paper of the ILAE commission for classification and terminology. Epilepsia 2017;58:512–521. 28276062 10.1111/epi.13709 PMC5386840

[8] WHOQOL – measuring quality of life| The World Health Organization. Available at: https://www.who.int/tools/whoqol (Accessed 18 November 2023).

[9] Baker GA Jacoby A Buck D. Quality of life of people with epilepsy: a European study. Epilepsia 1997;38:353–62. 9070599 10.1111/j.1528-1157.1997.tb01128.x

[10] Forsgren L Beghi E. Oun A. The epidemiology of epilepsy in Europe—a systematic review. Eur J Neurol 2005;12:245–53. 15804240 10.1111/j.1468-1331.2004.00992.x

[11] Jacoby A Baker GA. Quality-of-life trajectories in epilepsy: a review of the literature. Epilepsy Behav 2008;12:557–71. 18158270 10.1016/j.yebeh.2007.11.013

[12] Ronen GM Streiner DL Rosenbaum P. Health-related quality of life in childhood epilepsy: moving beyond control with minimal adverse effects. Health Qual Life Outcomes 2003;1:36. 14498989 10.1186/1477-7525-1-36 PMC201010

[13] Whitman S Hermann B. Psychopathology in Epilepsy: Social Dimensions. Oxford: Oxford University Press; 1986.

[14] Jennum P Gyllenborg J Kjellberg J. The social and economic consequences of epilepsy: a controlled national study. International League Against Epilepsy 2011;52:949–456. 10.1111/j.1528-1167.2010.02946.x 21275976

[15] Christensen J Vestergaard M Mortensen PB. Epilepsy and risk of suicide: a population-based case-control study. Lancet Neurol 2007;6:693–98. 17611160 10.1016/S1474-4422(07)70175-8

[16] Mutluay Fk Gunduz A Tekeoglu A. Health-related quality of life in patients with epilepsy in Turkey. J Phys Ther Sci 2016;28:240–45. 26957766 10.1589/jpts.28.240 PMC4756012

[17] Skevington SM Lotfy M. The World Health Organization’s WHOQOL-BREF quality of life assessment: psychometric properties and results of the international field trial: a Report from the WHOQOL Group. Journal of Research Gate 2004. 10.1023/B:QURE.0000018486.91360.00 15085902

[18] Guekht AB Mitrokhina TV Lebedeva AV. Factors influencing on quality of life in people with epilepsy. Seizure 2007;16:128–133. 17157536 10.1016/j.seizure.2006.10.011

[19] Shetty PH Naik RK Saroja AO. Quality of life in patients with epilepsy in India. J Neurosci Rural Pract 2011;02:033–038. 10.4103/0976-3147.80092 PMC3123012 21716845

[20] 36-Item Short Form Survey Instrument (SF-36), available from: https://www.rand.org/health-care/surveys_tools/mos/36-item-short-form/survey-instrument.html (Accessed August 2022).

[21] Brazier JE Harper R Jones NM. Validating the SF-36 health survey questionnaire: new outcome measure for primary care. Bmj 1992;305:160–64. 1285753 10.1136/bmj.305.6846.160 PMC1883187

[22] JE W Jr Gandek B. Overview of the SF-36 Health Survey and the International Quality of Life Assessment (IQOLA) Project. J Clin Epidemiol 1998;51:903–12. 9817107 10.1016/s0895-4356(98)00081-x

[23] 36-Item Short Form Survey (SF-36) Scoring Instructions [Internet]. RAND Corporation. 2019. Available from: https://www.rand.org/health-care/surveys_tools/mos/36-item-short-form/scoring.html (Accessed August 2022).

[24] Agha RA Mathew G Rashid R. Revised strengthening the reporting of cohort, cross-sectional and case-control studies in surgery (STROCSS) Guideline: an update for the age of artificial intelligence. Premier J Sci 2025;10:100081.

[25] Brülde B Malmgren K. Epilepsivårdens mål–en mångdimensionell modell. målbeskrivning grund för effektmätning [Goals of epilepsy treatment–a multidimensional model. The description of goals as a basis for efficiency measurement]. Lakartidningen 1999;96:3943–46. 10522104

[26] Baker GA Jacoby A Gorry J. Ellina V.Quality of life of people with epilepsy in Iran, the Gulf and Near East. The SIGN Study Epilepsia 2005;46:132–40. 15660779 10.1111/j.0013-9580.2005.20704.x

[27] Mosaku K Fatoye FO Komolafe M. Quality of life and associated factors among adults with epilepsy in Nigeria. Int J Psychiat Med 2006;36:469–81. 10.2190/R80G-580X-X1H2-6936 17408000

[28] Benamer HTS Grosset DG. A systematic review of the epidemiology of epilepsy in Arab countries. Epilepsia 2009;50:2301–04. 19389149 10.1111/j.1528-1167.2009.02058.x

[29] Jallon P. Epilepsy in developing countries. ILAE workshop report. Epilepsia 1997;38:1143–51. 9579962 10.1111/j.1528-1157.1997.tb01205.x

[30] Muche EA Ayalew MB Abdela OA. Assessment of quality of life of epileptic patients in ethiopia. Int J Chronic Dis 2020;2020:8714768. 31976314 10.1155/2020/8714768 PMC6961609

[31] El-Hilu SM. Social aspects of epilepsy in Kuwait. Int JSoc Psychiatry 1990;36:68–73. 2354888 10.1177/002076409003600108

[32] Sokrab M Sokrab A Elzubeir M. Quality of life in people with epilepsy in Sudan: an example of underserved communities in developing countries. Qatar Med J 2013;2012:59–63. 25003042 10.5339/qmj.2012.2.14 PMC3991048

[33] Abadiga M Mosisa G Amente T. Health-related quality of life and associated factors among epileptic patients on treatment follow up at public hospitals of Wollega zones, Ethiopia, 2018. BMC Res Notes 2019;12:679. 10.1186/s13104-019-4720-3 PMC6805515 31640789

[34] Mwangala PN Kariuki SM Nyongesa MK. Cognition, mood and quality-of-life outcomes among low literacy adults living with epilepsy in rural Kenya: a preliminary study. Epilepsy Behav 2018;85:45–51. 29908383 10.1016/j.yebeh.2018.05.032 PMC6086937

[35] Shakir M Al-Asadi JN. Quality of life and its determinants in people with epilepsy in Basrah, Iraq. Sultan QaboosUniv Med J 2012;12:449–57. PMC3523994 23275841

[36] Suurmeijer TP Reuvekamp MF Aldenkamp BP. Social functioning, psychological functioning, and quality of life in epilepsy. Epilepsia 2001;42:1160–68. 11580765 10.1046/j.1528-1157.2001.37000.x

[37] Wiebe S Bellhouse DR Fallahay C. Burden of epilepsy: the Ontario Health Survey. Can J Neurol Sci 1999;26:263–70. 10563210 10.1017/s0317167100000354

[38] Ohaeri JU Awadalla AW Farah AA. Quality of life in people with epilepsy and their family caregivers. An Arab experience using the short version of the World Health Organization quality of life instrument. Saudi Med J 2009;30:1328–35. 19838443

[39] Siebenbrodt K. ‘Determinants of quality of life in adults with epilepsy: a multicenter, cross-sectional study from Germany. Neurol. Res. Pract 2023;5:41. 37533112 10.1186/s42466-023-00265-5 PMC10398956

[40] Choi-Kwon S Chung C Kim H. Factors affecting the quality of life in patients with epilepsy in Seoul, South Korea. Acta Neurol Scand 2003;108:428–434. 14616296 10.1046/j.1600-0404.2003.00151.x

[41] Laccheo I Ablah E Heinrichs R. Assessment of quality of life among the elderly with epilepsy. Epilepsy & Behavior 2008;12:257–261. 17988950 10.1016/j.yebeh.2007.09.003

[42] Baker G Jacoby A Buck D. The quality of life of older people with epilepsy: findings from a UK community study. Seizure 2001;10:92–99. 11407951 10.1053/seiz.2000.0465

[43] Baranowski CJ. The quality of life of older adults with epilepsy: a systematic review. Seizure Eur J Epilepsy [Internet] 2018;60:190–97. 10.1016/j.seizure.2018.06.002 30031296

[44] Shetty PH Naik RK Saroja A. Quality of life in patients with epilepsy in India. J Neurosci Rural Pract 2011;2:33–38. 21716845 10.4103/0976-3147.80092 PMC3123012

[45] Huntington C Stanley SM Doss BD. ‘Happy, healthy, and wedded? How the transition to marriage affects mental and physical health. J. Fam. Psychol 2022;36:608–17. 34472934 10.1037/fam0000913 PMC8888778

